# Supplementary material for: Single-cell transcriptomic analyses of tumor microenvironment and molecular reprograming landscape of metastatic laryngeal squamous cell carcinoma
Source: Commun Biol. 2024 Jan 8;7:63. doi: 10.1038/s42003-024-05765-x (PMC10774275; doi:10.1038/s42003-024-05765-x)
Supplement: Supplementary file 2 — Supplementary Information [file 42003_2024_5765_MOESM2_ESM.pdf]

Supplementary Figures

Single-Cell Transcriptomic Analyses of Tumor Microenvironment and Molecular Reprograming Landscape of Metastatic Laryngeal Squamous Cell Carcinoma

Yuanyuan Sun[1], Sheng Chen[2], Yongping Lu[3], Zhenming Xu[4,*], Weineng Fu[1,*], Wei Yan[5,6,*]

[1]Department of Medical Genetics, China Medical University, Shenyang, 110122, China

[2]Department of Laboratory Animal Science, China Medical University, Shenyang, 110122, China

[3]NHC Key Laboratory of Reproductive Health and Medical Genetics, Shenyang, 110122, China

[4]Department of Otolaryngology, the Fourth People's Hospital of Shenyang City, Shenyang, 110031, China

[5]The Lundquist Institute for Biomedical Innovation at Harbor-UCLA Medical Center, Torrance, CA 90502, USA

[6]Department of Medicine, David Geffen School of Medicine at UCLA, Los Angeles, CA 90095, USA

*Correspondence

wei.yan@lundquist.org or wnfu@cmu.edu.cn or zhenmingxu@cmu.edu.cn

This file contains 11 figures (Figure S1-S11) and 3 tables (Table S1-S3).

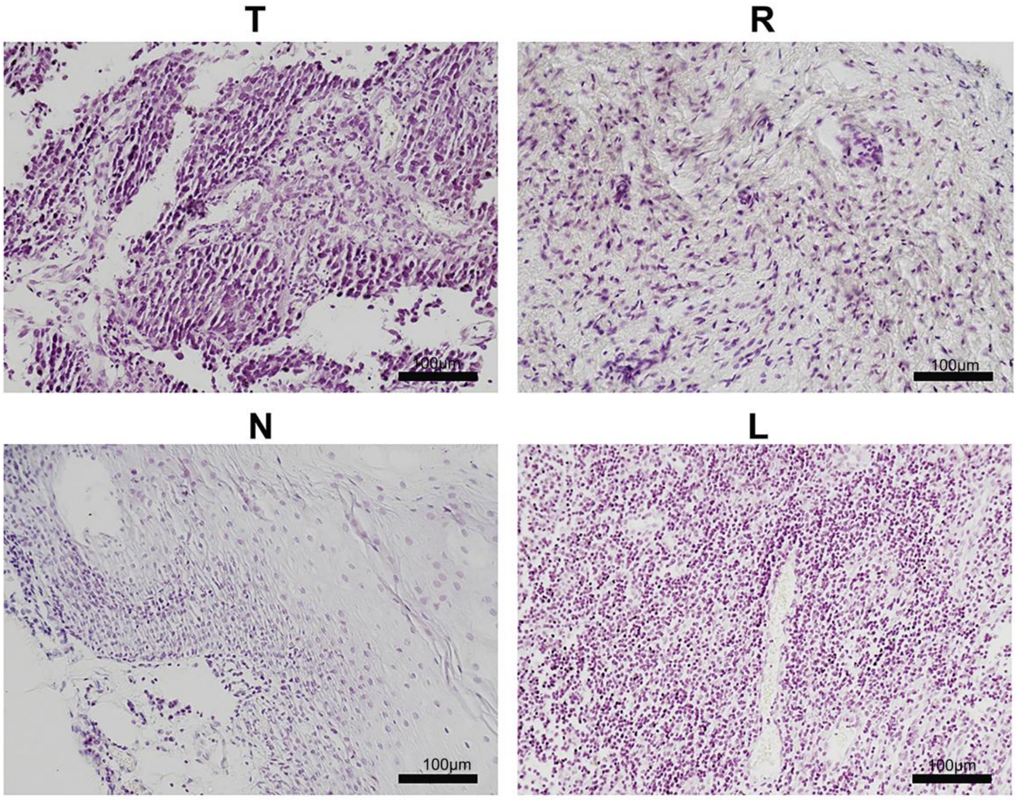

Figure S1. Histology of the four types of tissues collected from LSCC patients for scRNA-seq analyses, including LSCC *in situ* (T), normal laryngeal mucosal epithelia adjacent to the cancerous tissue (N), tissue from the LSCC margin (R), and the lymph node with metastasized LSCC (L). Scale bars = 100μm.

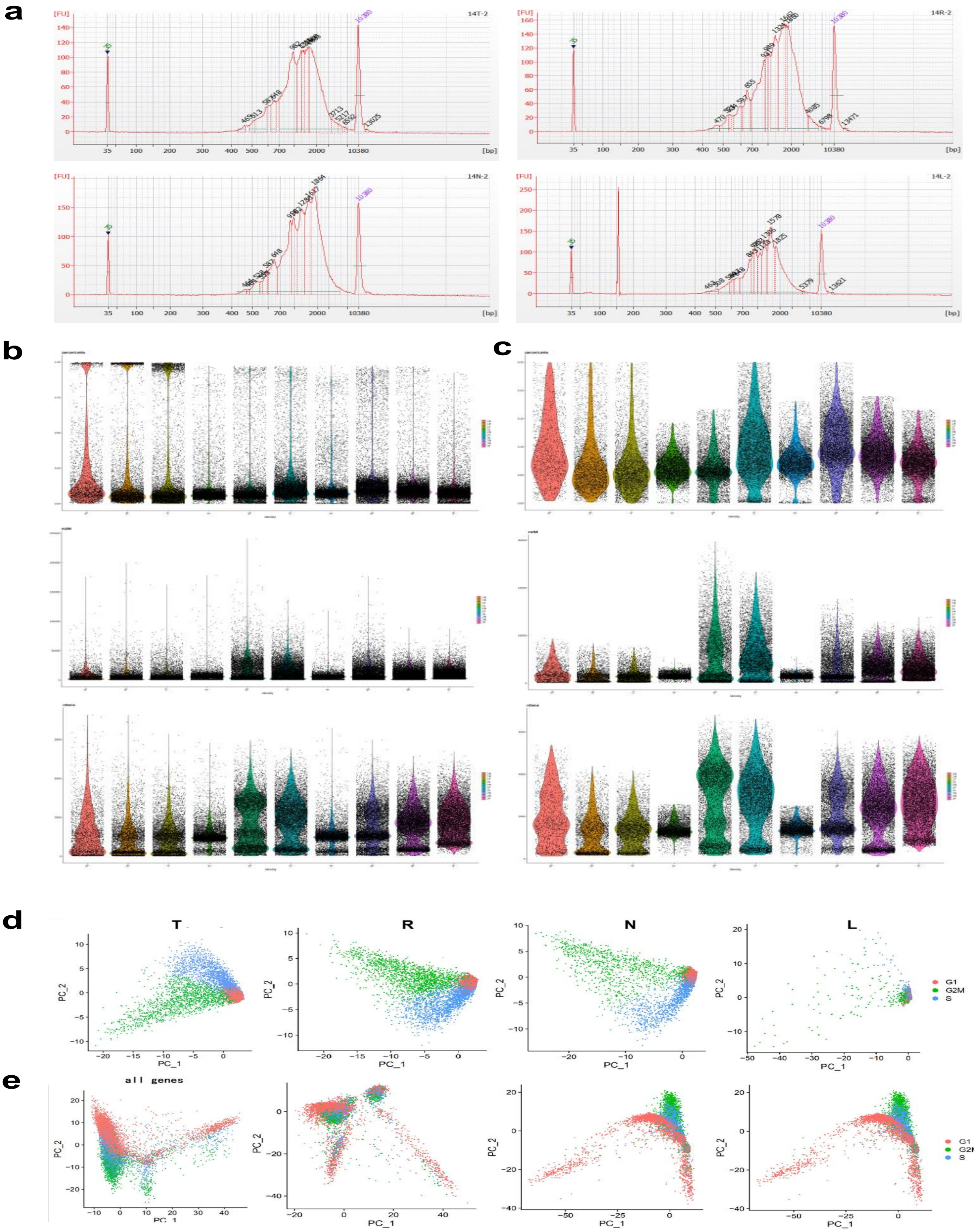

Figure S2. Quality control and cell filtering of sc-RNA seq data. a. Size distribution of the sc-RNA seq cDNA library detected by Agilent 2100 Bioanalyzer. b. Percentage distribution of the mitochondrial genes, unique molecular identifier (UMI), and genes before filtering. c. Distribution of percent of mitochondria genes, UMI, and genes after filtering. d. PCA visualizes to assess the affect of cell cycle-related genes on clustering. e. PCA visualizes to show cell clusters based on all genes expressed in cells.

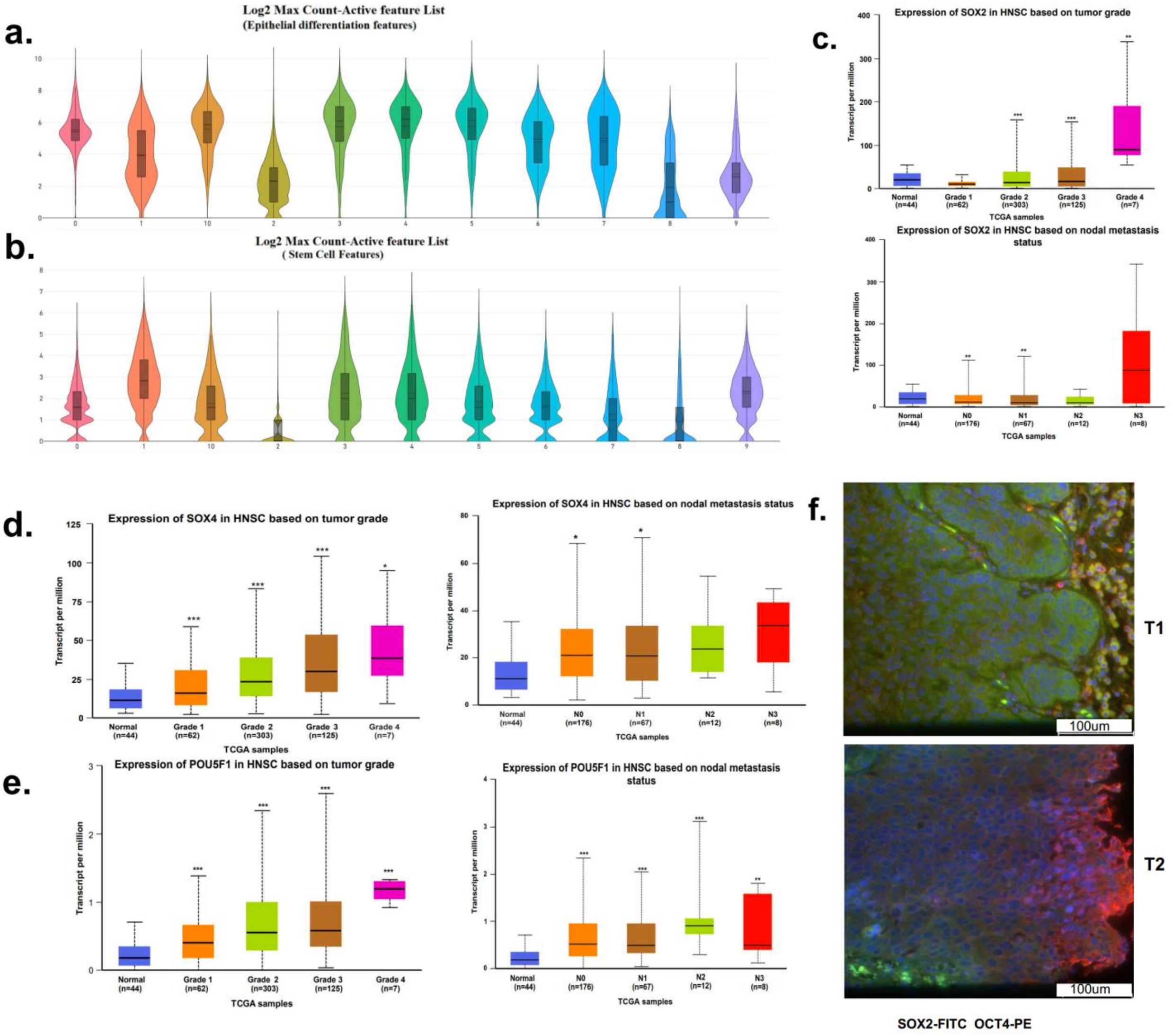

Figure S3. Stem cell feature in malignant cells are responsible for the invasion and metastasis of LSCC. a. Violin plots showing the expression of stem features (*SOX2, SOX4, OCT4*) among epithelial-derived cell subclusters. b. Violin plots showing the expression of epithelial features (*KRT1, KRT 5, KRT10, KRT14, KRT15, KRT16*) among epithelial-derived cell subclusters. c. The relationship of the expression of stem features *SOX2,* and head and neck squamous cell cancer (HNSCC) progression. d. The relationship of the expression of stem features *SOX4* and head and neck squamous cell cancer (HNSCC) progression. e. The relationship of the expression of stem features *OCT4* and head and neck squamous cell cancer (HNSCC) progression. f. The expression of stem features (*SOX2* and *OCT4*) in laryngeal squamous cell cancer (LSCC) with or without metastasis.

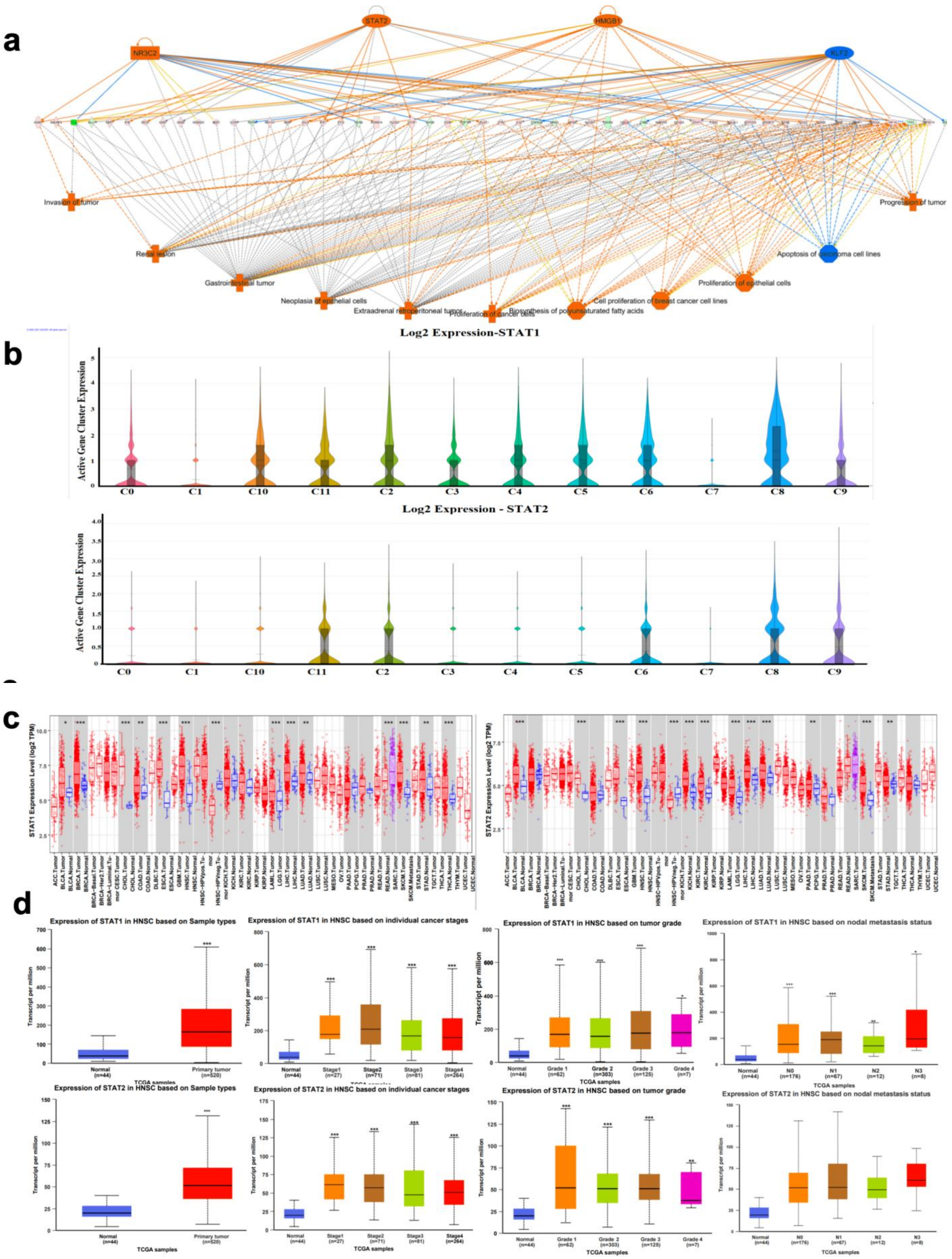
a
STAT2
HMGB1
NR3C2
KLF2
Invasion of tumor
Progression of tumor
Renal lesion
Apoptosis of carcinoma cell lines
Gastrointestinal tumor
Proliferation of epithelial cells
Neoplasia of epithelial cells
Cell proliferation of breast cancer cell lines
Extraadrenal retroperitoneal tumor
Proliferation of cancer cells
Biosynthesis of polyunsaturated fatty acids
b
Log2 Expression-STAT1
Active Gene Cluster Expression
C0 C1 C10 C11 C2 C3 C4 C5 C6 C7 C8 C9
Log2 Expression - STAT2
Active Gene Cluster Expression
C0 C1 C10 C11 C2 C3 C4 C5 C6 C7 C8 C9
c
STAT1 Expression Level (log2 TPM)
STAT2 Expression Level (log2 TPM)
d
Expression of STAT1 in HNSC based on Sample types
Expression of STAT1 in HNSC based on individual cancer stages
Expression of STAT1 in HNSC based on tumor grade
Expression of STAT1 in HNSC based on nodal metastasis status
Expression of STAT2 in HNSC based on Sample types
Expression of STAT2 in HNSC based on individual cancer stages
Expression of STAT2 in HNSC based on tumor grade
Expression of STAT2 in HNSC based on nodal metastasis status
Transcript per million
TCGA samples
Normal (n=44)
Primary tumor (n=520)
Stage1 (n=27)
Stage2 (n=71)
Stage3 (n=81)
Stage4 (n=264)
Grade 1 (n=62)
Grade 2 (n=303)
Grade 3 (n=125)
Grade 4 (n=7)
N0 (n=176)
N1 (n=67)
N2 (n=12)
N3 (n=8)

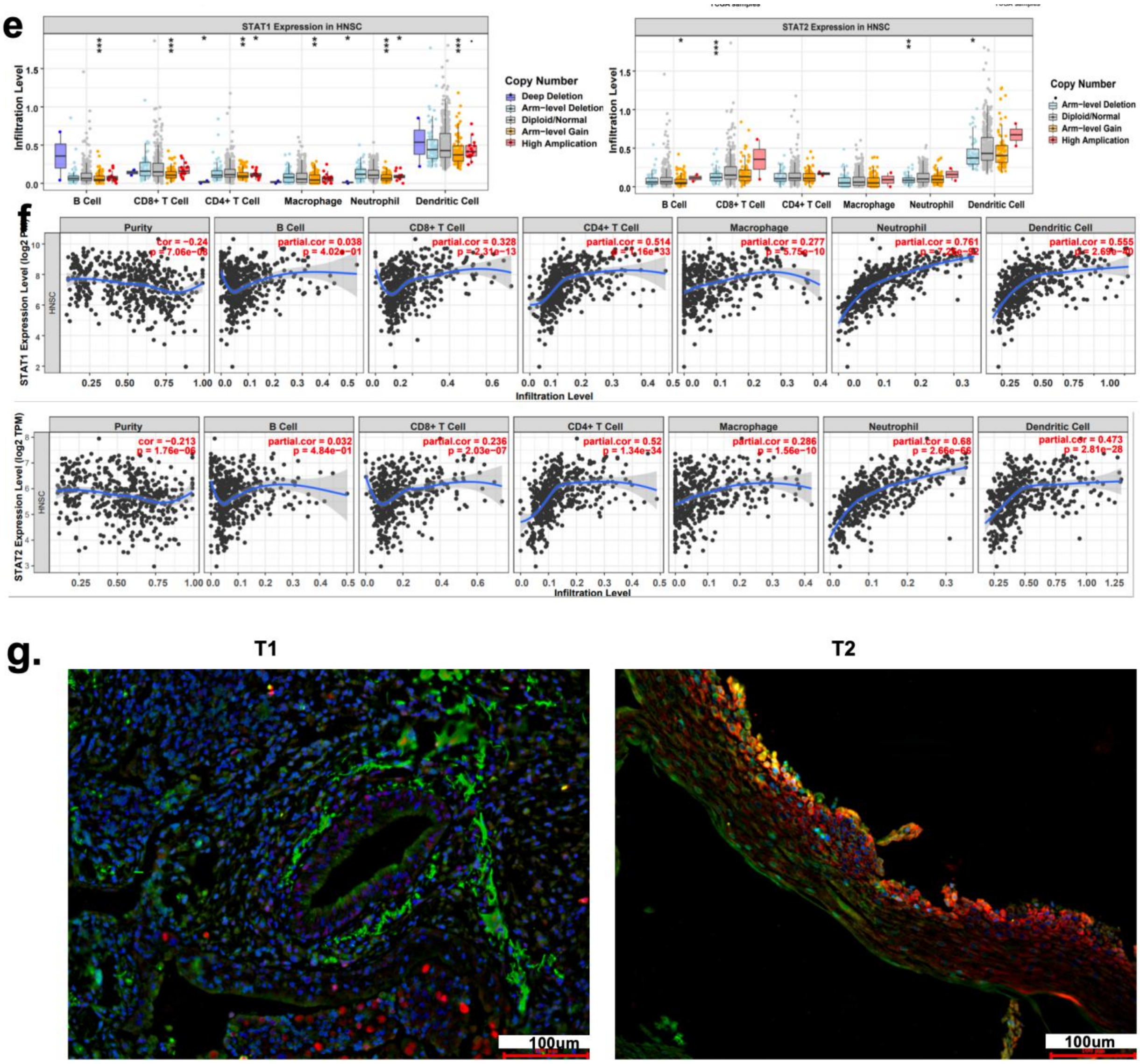

STAT1-FITC and STAT2-PE

Figure S4. *STAT1* and *STAT2* play important roles in HNSCC progression. a. Interaction and regulatory networks in epithelial-derived cells. b. *STAT1/2* expression in each of the epithelial-derived cell subclusters in LSCC. c. *STAT1/2* expression levels in various cancer types. d. The relationship between STAT1/2 expression levels and cancer grades, stages and nodal metastasis in HNSCC. e. Box plots showing relationship between copy number variations in *STAT1* and *STAT2* and immune cell infiltration levels in HNSCC. SCNAs are defined by GISTIC 2.0, including deep deletion (-2), arm-level deletion (-1), diploid/normal (0), arm-level gain (1), and high amplification. f. The relationship between *STAT1/2* expression levels and tumor immune infiltration levels in HNSCC. g. The expression of STAT1 and STAT2 in laryngeal squamous cell cancer (LSCC) with or without metastasis.

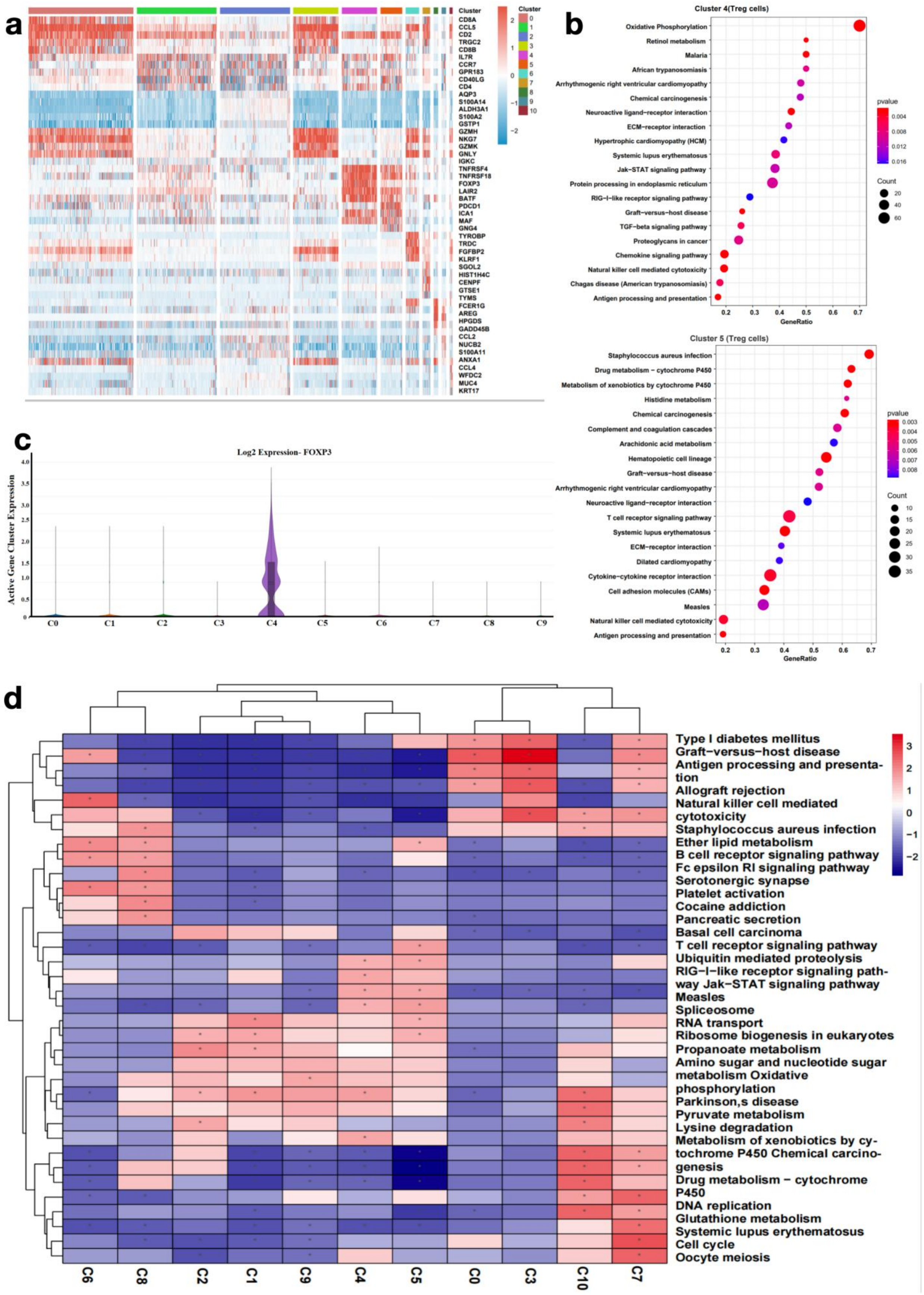
a
Cluster
CD8A
CCL5
CD2
TRGC2
CD8B
IL7R
CCR7
GPR183
CD40LG
CD4
AQP3
S100A14
ALDH3A1
S100A2
GSTP1
GZMH
NKG7
GZMK
GNLY
IGKC
TNFRSF4
TNFRSF18
FOXP3
LAIR2
BATF
PDCD1
ICA1
MAF
GNG4
TYROBP
TRDC
FGFBP2
KLRF1
SGOL2
HIST1H4C
CENPF
GTSE1
TYMS
FCER1G
AREG
HPGDS
GADD45B
CCL2
NUCB2
S100A11
ANXA1
CCL4
WFDC2
MUC4
KRT17
b
Cluster 4(Treg cells)
Oxidative Phosphorylation
Retinol metabolism
Malaria
African trypanosomiasis
Arrhythmogenic right ventricular cardiomyopathy
Chemical carcinogenesis
Neuroactive ligand−receptor interaction
ECM−receptor interaction
Hypertrophic cardiomyopathy (HCM)
Systemic lupus erythematosus
Jak−STAT signaling pathway
Protein processing in endoplasmic reticulum
RIG−I−like receptor signaling pathway
Graft−versus−host disease
TGF−beta signaling pathway
Proteoglycans in cancer
Chemokine signaling pathway
Natural killer cell mediated cytotoxicity
Chagas disease (American trypanosomiasis)
Antigen processing and presentation
GeneRatio
pvalue
Count
Cluster 5 (Treg cells)
Staphylococcus aureus infection
Drug metabolism − cytochrome P450
Metabolism of xenobiotics by cytochrome P450
Histidine metabolism
Chemical carcinogenesis
Complement and coagulation cascades
Arachidonic acid metabolism
Hematopoietic cell lineage
Graft−versus−host disease
Arrhythmogenic right ventricular cardiomyopathy
Neuroactive ligand−receptor interaction
T cell receptor signaling pathway
Systemic lupus erythematosus
ECM−receptor interaction
Dilated cardiomyopathy
Cytokine−cytokine receptor interaction
Cell adhesion molecules (CAMs)
Measles
Natural killer cell mediated cytotoxicity
Antigen processing and presentation
GeneRatio
pvalue
Count
c
Log2 Expression- FOXP3
Active Gene Cluster Expression
C0
C1
C2
C3
C4
C5
C6
C7
C8
C9
d
Type I diabetes mellitus
Graft−versus−host disease
Antigen processing and presentation
Allograft rejection
Natural killer cell mediated cytotoxicity
Staphylococcus aureus infection
Ether lipid metabolism
B cell receptor signaling pathway
Fc epsilon RI signaling pathway
Serotonergic synapse
Platelet activation
Cocaine addiction
Pancreatic secretion
Basal cell carcinoma
T cell receptor signaling pathway
Ubiquitin mediated proteolysis
RIG−I−like receptor signaling pathway Jak−STAT signaling pathway
Measles
Spliceosome
RNA transport
Ribosome biogenesis in eukaryotes
Propanoate metabolism
Amino sugar and nucleotide sugar metabolism Oxidative phosphorylation
Parkinson,s disease
Pyruvate metabolism
Lysine degradation
Metabolism of xenobiotics by cytochrome P450 Chemical carcinogenesis
Drug metabolism − cytochrome P450
DNA replication
Glutathione metabolism
Systemic lupus erythematosus
Cell cycle
Oocyte meiosis
C6
C8
C2
C1
C9
C4
C5
C0
C3
C10
C7

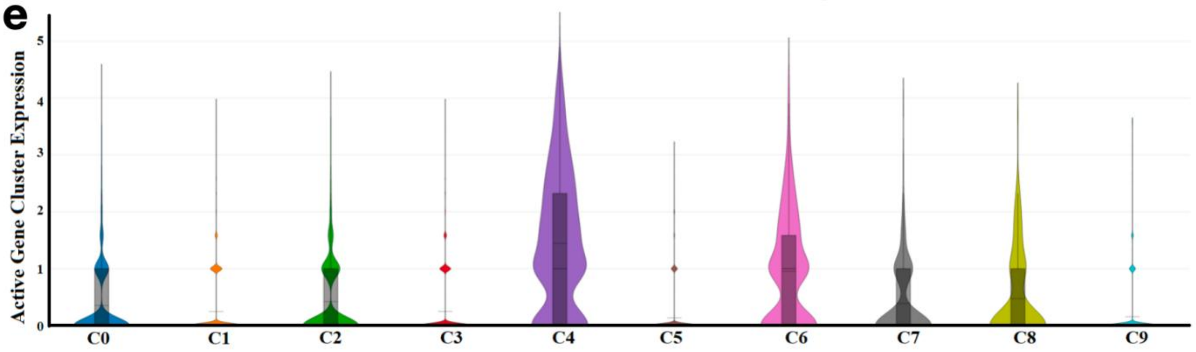

Figure S5. Function enrichment analyses of the T cell subclusters. a. Heatmap showed the top 5 most significant DEGs among the eleven T cell subclusters. b. The significantly activated pathways in the regulatory T cells (Tregs) (C4, C5). c. Violin plots showing the levels of *FOXP3* among the eleven T cell subclusters. d.GSEA results showing enriched biological function GO terms among the eleven T cell subclusters. e.Violin plots showing the levels of *BATF* among the eleven T cell subclusters.

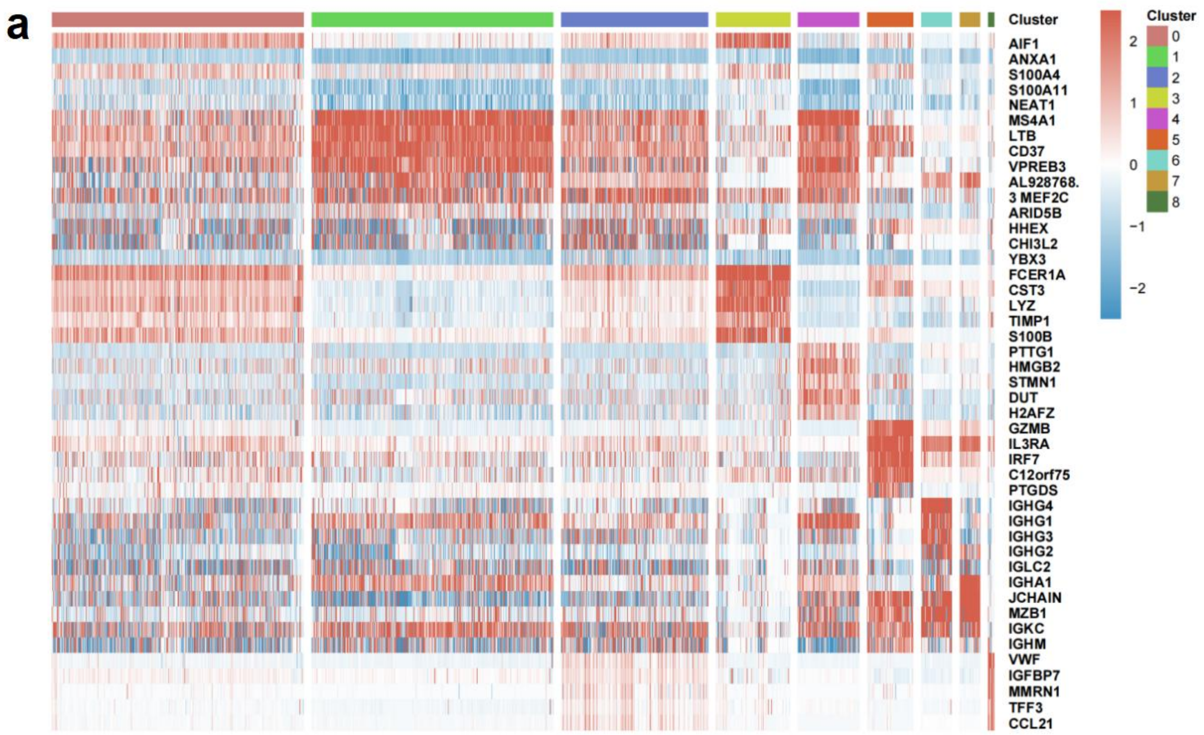

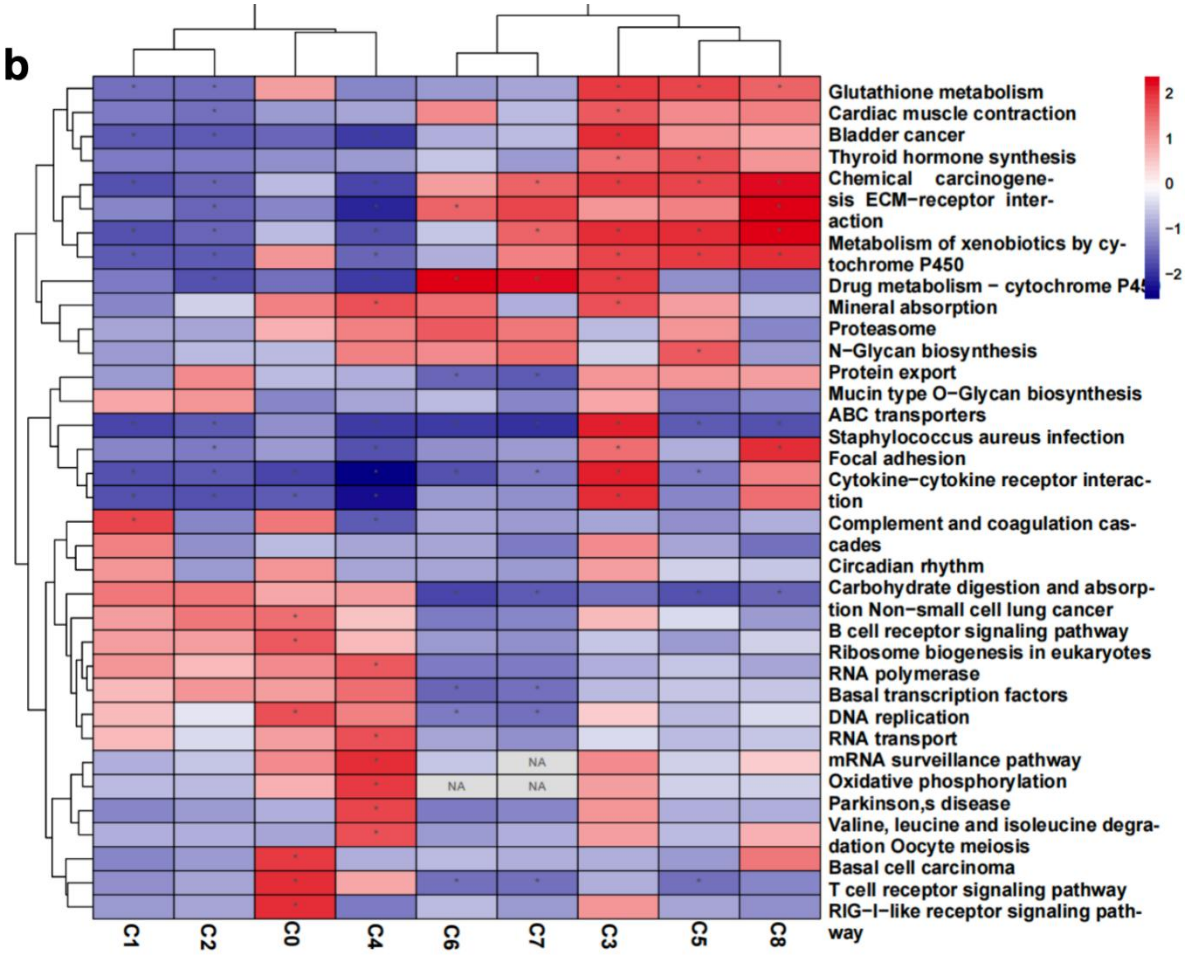
b
Glutathione metabolism
Cardiac muscle contraction
Bladder cancer
Thyroid hormone synthesis
Chemical carcinogenesis
ECM−receptor interaction
Metabolism of xenobiotics by cytochrome P450
Drug metabolism − cytochrome P450
Mineral absorption
Proteasome
N−Glycan biosynthesis
Protein export
Mucin type O−Glycan biosynthesis
ABC transporters
Staphylococcus aureus infection
Focal adhesion
Cytokine−cytokine receptor interaction
Complement and coagulation cascades
Circadian rhythm
Carbohydrate digestion and absorption
Non−small cell lung cancer
B cell receptor signaling pathway
Ribosome biogenesis in eukaryotes
RNA polymerase
Basal transcription factors
DNA replication
RNA transport
mRNA surveillance pathway
Oxidative phosphorylation
Parkinson,s disease
Valine, leucine and isoleucine degradation
Oocyte meiosis
Basal cell carcinoma
T cell receptor signaling pathway
RIG−I−like receptor signaling pathway
C1
C2
C0
C4
C6
C7
C3
C5
C8
NA
2
1
0
−1
−2

c

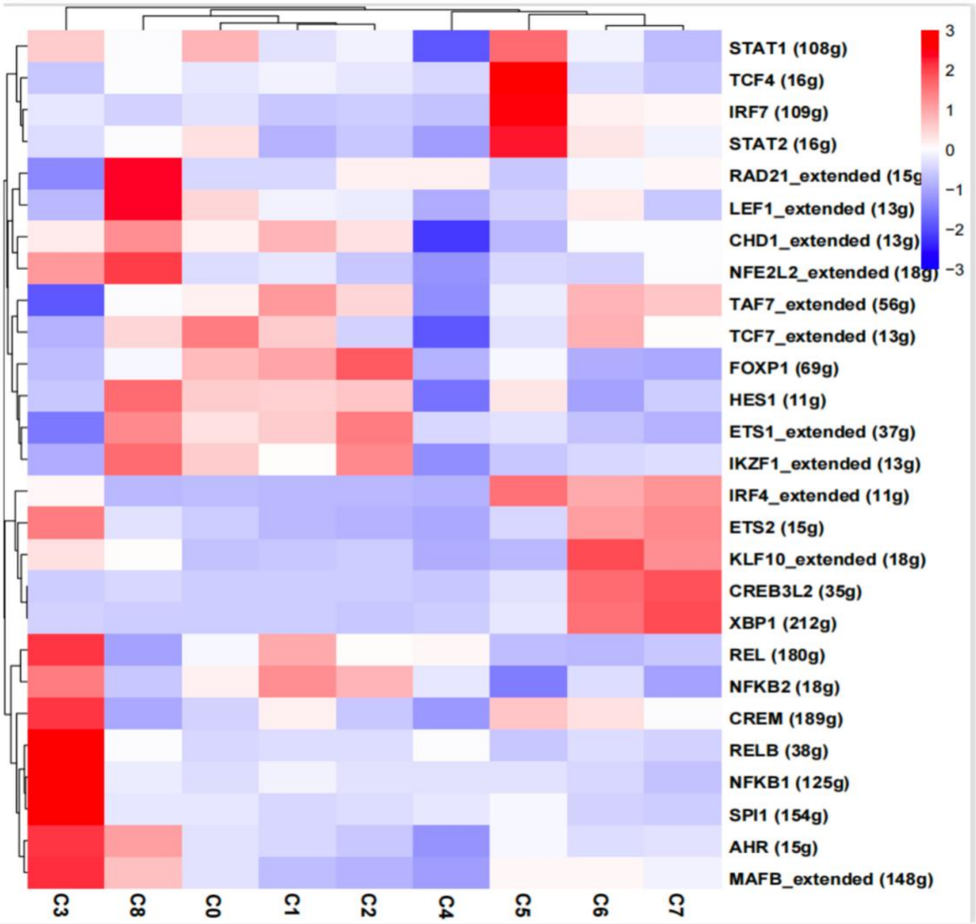

Figure S6. Function enrichment analyses of the nine B cell subclusters. a. Heatmap showing the top 5 most significant DEGs among the nine B cell subclusters. b. GSEA results showing enriched bio-function GO terms among the nine B cell subclusters. c. Heatmap showing the activity of transcription factors (TFs) among the nine B cell subclusters by SCENIC analyses.

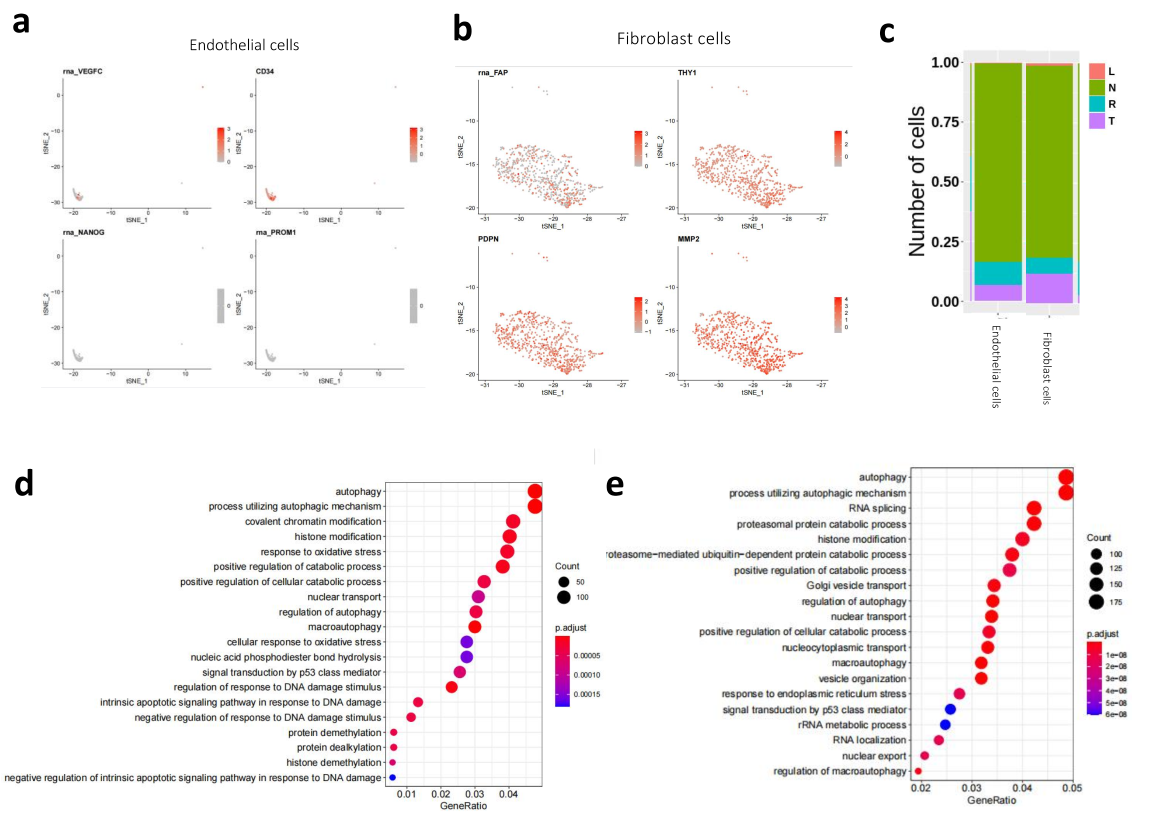

Figure S7. Stromal cell autophagy in tumor microenvironment promotes invasion and metastasis of laryngeal carcinoma. a t-SNE plots showing marker gene expression in normal endothelial cells. b. t-SNE plots showing marker gene expression in normal fibroblast cells. c. The relative abundance of endothelial and fibroblast cells in the four types of tissues analyzed in the present study (L, N, R, T). d. GSEA results showing activated pathways in endothelial cells of LSCC. d. GSEA results showing activated pathways in fibroblast cells of LSCC.

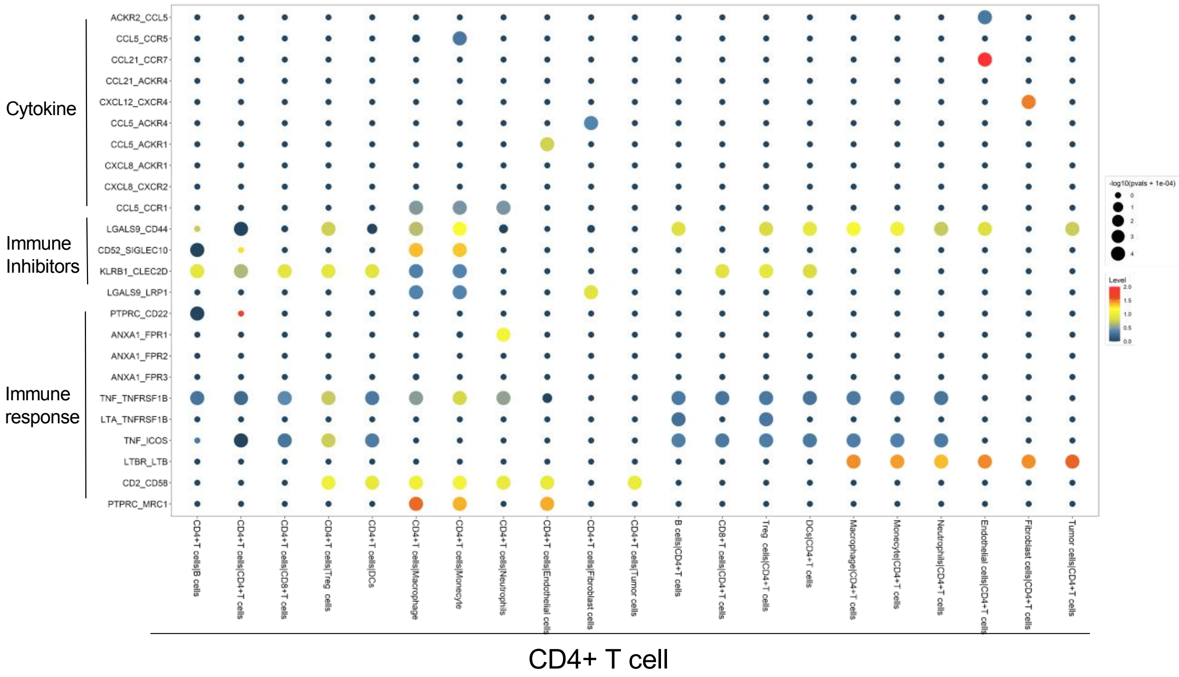

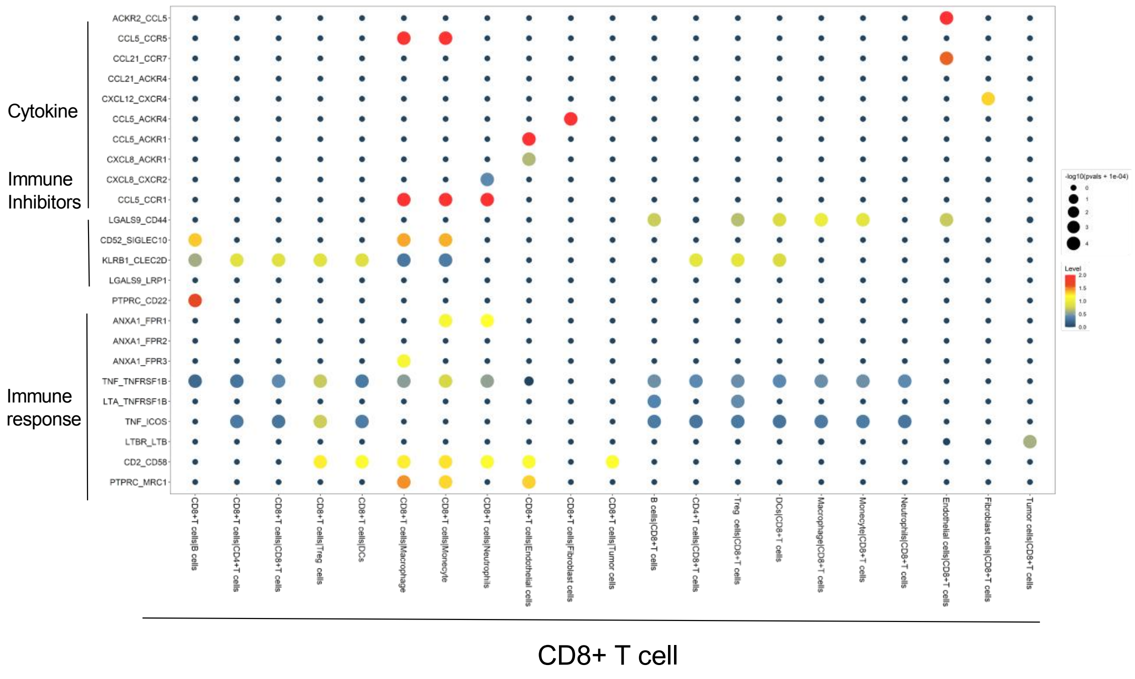

Figure S8. Summary of selected ligand-receptor interactions between CD4+ T cells and other cell types or between CD8+ T cells and other cell types in LSCC TME.

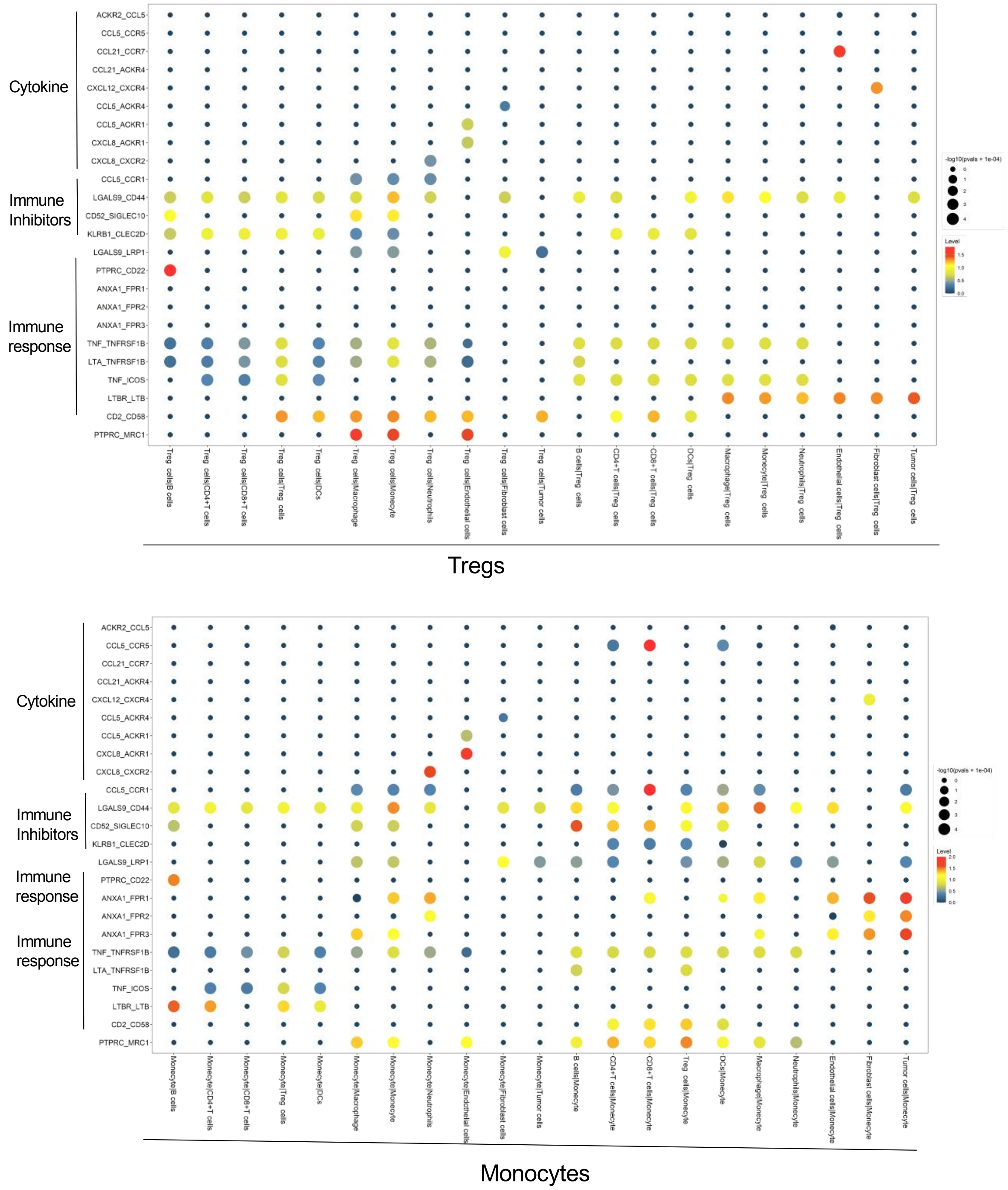

Figure S9. Summary of selected ligand-receptor interactions between Tregs and other cell types or between monocytes and other cell types in LSCC TME.

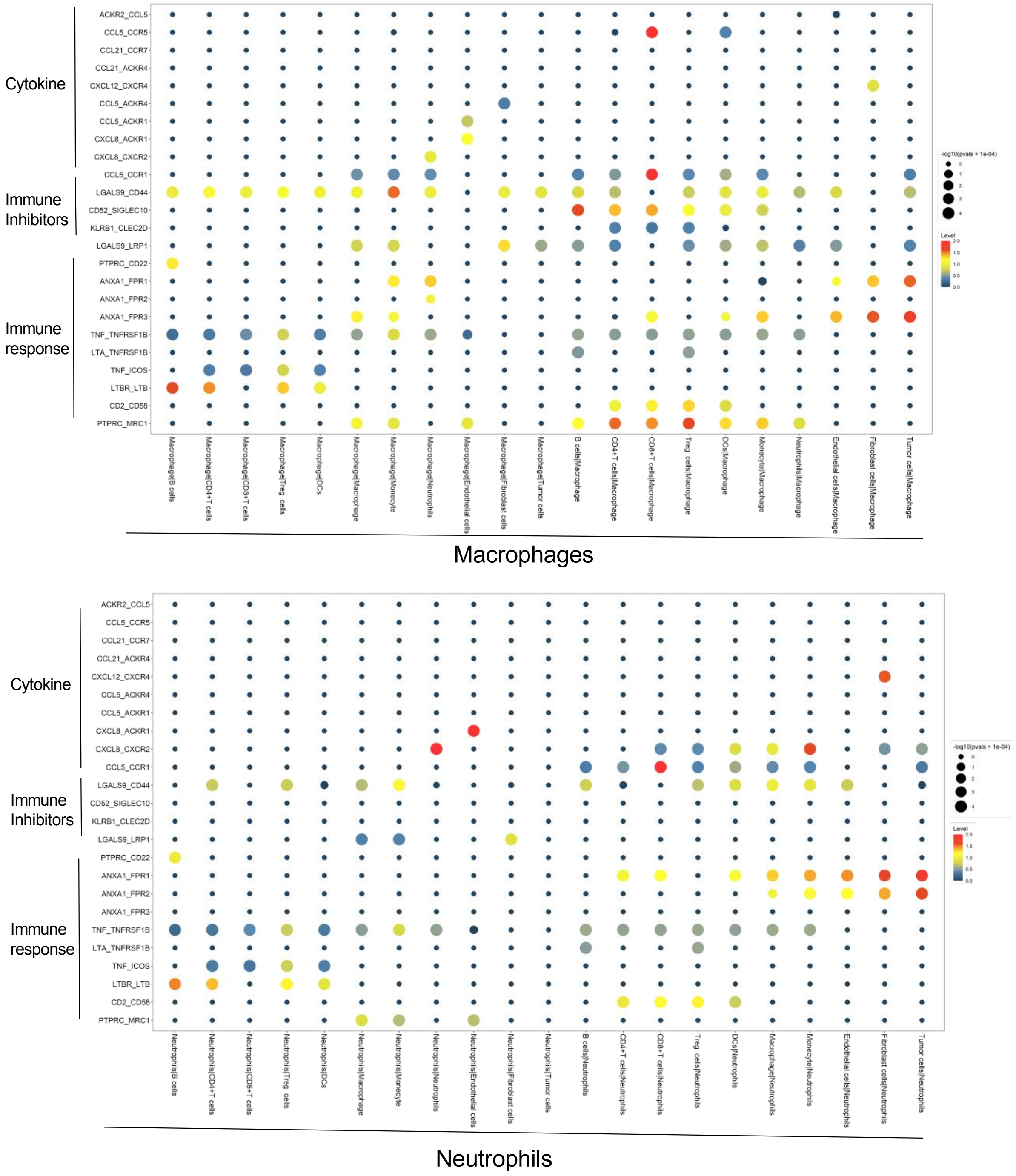

Figure S10. Summary of selected ligand-receptor interactions between macrophages and other cell types or between neutrophils and other cell types in LSCC TME.

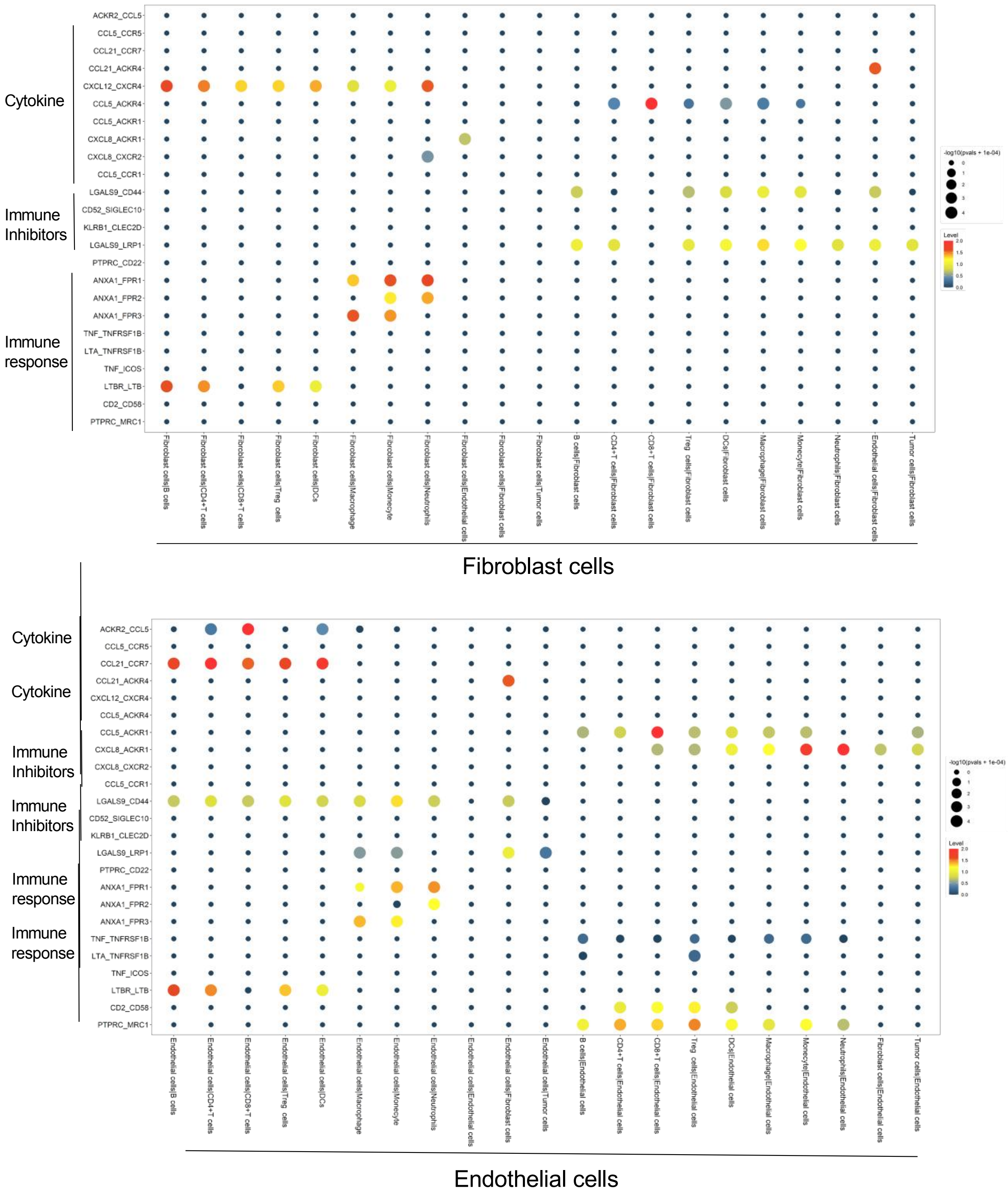

Figure S11. Summary of selected ligand-receptor interactions between fibroblast cells and other cell types or between endothelial cells and other cell types in LSCC TME.

Table S1. Patient information.

Patient ID	Age	Gender	Stages	Lesions	Tumor type	Lymphatic invasion	Distant metastasis
1	61	Male	T3N1M0	T, R, N	Supraglottic LSCC	invasion	non
2	65	Male	T3N2M0	T,N,L	Supraglottic LSCC	invasion	non
3	63	Male	T3N2M0	T, R, N, L	Supraglottic LSCC	invasion	non
4	53	Male	T2N0M0	T	Supraglottic LSCC	non	non
5	72	Male	T1N0M1	T	Supraglottic LSCC	non	non
6	68	Male	T2N0M2	T	Supraglottic LSCC	non	non

Table S2. Summary of 10X scRNA-seq data.

Sample	Actual Cell#	Medium Gene#	Cell# post filtering	Medium Gene# Post Filtering
1T	7,320	1,345	4,561	1,352
1R	5,630	1,192	3,605	1,223
1N	2,564	1,581	1,501	1,803
2T	10,009	2,883	8,101	2,904
2L	8,218	1,496	6,299	1,347
2N	9,223	2,593	7,305	3,075
3T	11,375	2,716	10,839	2,384
3R	12,796	2,515	9,488	2,665
3L	7,749	1,519	5,679	1,400
3N	11,590	1,923	9,185	1,951
T2-2	10,358	1,604	7,475	2,784
T2-3	11,379	2,237	9,137	2,646
T2-4	6,948	1,104	5,444	1,724
**Total**			88619	

Table S3. GO and KEGG analyses in EpCs.

	go	kegg
0	epidermis development	Staphylococcus aureus infection
	skin development	Phagosome
	regulation of peptidase activity	p53 signaling pathway
	negative regulation of proteolysis	Rheumatoid arthritis

	collagen-containing extracellular matrix	Viral myocarditis
1	epidermis development	Pathways in cancer
	skin development	p53 signaling pathway
	neutrophil activation	Phagosome
	regulation of peptidase activity	Rheumatoid arthritis
	epidermal cell differentiation	Cell cycle
2	skin development	Cell cycle
	epidermis development	Phagosome
	collagen-containing extracellular matrix	Staphylococcus aureus infection
	cell adhesion molecule binding	p53 signaling pathway
	regulation of peptidase activity	Cell adhesion molecules (CAMs)
3	epidermis development	Gap junction
	regulation of peptidase activity	Oocyte meiosis
	skin development	Cell cycle
	epidermal cell differentiation	Cytokine-cytokine receptor interaction
	negative regulation of proteolysis	Glutathione metabolism
4	nuclear division	Cell cycle
	organelle fission	DNA replication
	DNA replication	p53 signaling pathway
	positive regulation of cell cycle	Pyrimidine metabolism
	chromosomal region	Oocyte meiosis
5	nuclear division	Cell cycle
	organelle fission	Oocyte meiosis
	chromosome segregation	Progesterone-mediated oocyte maturation
	mitotic nuclear division	p53 signaling pathway
	spindle	Pyrimidine metabolism
6	collagen-containing extracellular matrix	Focal adhesion
	extracellular structure organization	ECM-receptor interaction
	extracellular matrix organization	Cytokine-cytokine receptor interaction

	epidermis development	Rheumatoid arthritis
	skin development	Amoebiasis
7	epidermis development	Phagosome
	skin development	Rheumatoid arthritis
	epidermal cell differentiation	Cytokine-cytokine receptor interaction
	leukocyte migration	Antigen processing and presentation
	keratinocyte differentiation	Hematopoietic cell lineage
8	epidermis development	p53 signaling pathway
	skin development	Cell cycle
	neutrophil degranulation	Pathways in cancer
	neutrophil activation involved in immune response	Phagosome
	neutrophil activation	Cytokine-cytokine receptor interaction
9	epidermis development	Cell cycle
	skin development	Pathways in cancer
	cell-cell junction	Phagosome
	neutrophil activation	p53 signaling pathway
	epithelial cell proliferation	Rheumatoid arthritis
10	epidermis development	Toxoplasmosis
	neutrophil activation	Cell adhesion molecules (CAMs)
	collagen-containing extracellular matrix	Phagosome
	regulation of cell-cell adhesion	Rheumatoid arthritis
	skin development	Staphylococcus aureus infection
11	collagen-containing extracellular matrix	Pathways in cancer
	epidermis development	Focal adhesion
	skin development	Rheumatoid arthritis
	epithelial cell proliferation	Cytokine-cytokine receptor interaction
	cell-cell junction	p53 signaling pathway
