# Supplementary material for: Single-cell transcriptomic analyses of tumor microenvironment and molecular reprograming landscape of metastatic laryngeal squamous cell carcinoma
Source: Commun Biol. 2024 Jan 8;7:63. doi: 10.1038/s42003-024-05765-x (PMC10774275; doi:10.1038/s42003-024-05765-x)
Supplement: Supplementary file 5 — Reporting Summary [file 42003_2024_5765_MOESM5_ESM.pdf]

nature portfolio

Corresponding author(s): COMMSBIO-22-4201-T

Last updated by author(s): Oct 30, 2013

Reporting Summary

Nature Portfolio wishes to improve the reproducibility of the work that we publish. This form provides structure for consistency and transparency in reporting. For further information on Nature Portfolio policies, see our Editorial Policies and the Editorial Policy Checklist.

Statistics

For all statistical analyses, confirm that the following items are present in the figure legend, table legend, main text, or Methods section.

n/a	Confirmed	
☐	☒	The exact sample size (*n*) for each experimental group/condition, given as a discrete number and unit of measurement
☐	☒	A statement on whether measurements were taken from distinct samples or whether the same sample was measured repeatedly
☐	☒	The statistical test(s) used AND whether they are one- or two-sided<br>*Only common tests should be described solely by name; describe more complex techniques in the Methods section.*
☒	☐	A description of all covariates tested
☐	☒	A description of any assumptions or corrections, such as tests of normality and adjustment for multiple comparisons
☒	☐	A full description of the statistical parameters including central tendency (e.g. means) or other basic estimates (e.g. regression coefficient) AND variation (e.g. standard deviation) or associated estimates of uncertainty (e.g. confidence intervals)
☐	☒	For null hypothesis testing, the test statistic (e.g. *F*, *t*, *r*) with confidence intervals, effect sizes, degrees of freedom and *P* value noted<br>*Give P values as exact values whenever suitable.*
☐	☒	For Bayesian analysis, information on the choice of priors and Markov chain Monte Carlo settings
☐	☒	For hierarchical and complex designs, identification of the appropriate level for tests and full reporting of outcomes
☐	☒	Estimates of effect sizes (e.g. Cohen's *d*, Pearson's *r*), indicating how they were calculated

Our web collection on statistics for biologists contains articles on many of the points above.

Software and code

Policy information about availability of computer code

Data collection	R version 4.0.2 (2020-06-22)
Data analysis	Louvain algorithm, SCENIC, CNV analyses, cellchat

For manuscripts utilizing custom algorithms or software that are central to the research but not yet described in published literature, software must be made available to editors and reviewers. We strongly encourage code deposition in a community repository (e.g. GitHub). See the Nature Portfolio guidelines for submitting code & software for further information.

Data

Policy information about availability of data

All manuscripts must include a data availability statement. This statement should provide the following information, where applicable:

- Accession codes, unique identifiers, or web links for publicly available datasets
- A description of any restrictions on data availability
- For clinical datasets or third party data, please ensure that the statement adheres to our policy

GES206332

Research involving human participants, their data, or biological material

Policy information about studies with human participants or human data. See also policy information about sex, gender (identity/presentation), and sexual orientation and race, ethnicity and racism.

Reporting on sex and gender	male
Reporting on race, ethnicity, or other socially relevant groupings	Asian
Population characteristics	China
Recruitment	Laryngeal squamous cell carcinoma (LSCC) in situ (T), normal laryngeal mucosal epithelial tissue adjacent to LSCC (N), marginal tissue of LSCC (R), and lymph nodes with metastatic LSCC (L) from 6 LSCC patients with or without lymphatic metastasis were collected from the Department of Otolaryngology, the Fourth People's Hospital of Shenyang City.
Ethics oversight	All the patients gave their informed consent and the study was approved by the Institutional Review Board of China Medical University, in accordance with the Declaration of Helsinki.

Note that full information on the approval of the study protocol must also be provided in the manuscript.

Field-specific reporting

Please select the one below that is the best fit for your research. If you are not sure, read the appropriate sections before making your selection.

☒ Life sciences ☐ Behavioural & social sciences ☐ Ecological, evolutionary & environmental sciences

For a reference copy of the document with all sections, see nature.com/documents/nr-reporting-summary-flat.pdf

Life sciences study design

All studies must disclose on these points even when the disclosure is negative.

Sample size	Simple random sampling.
Data exclusions	No datas were excluded.
Replication	All attempts at replication were successful.
Randomization	Random sampling.
Blinding	LSCC with or without lymphatic metastasis.

Reporting for specific materials, systems and methods

We require information from authors about some types of materials, experimental systems and methods used in many studies. Here, indicate whether each material, system or method listed is relevant to your study. If you are not sure if a list item applies to your research, read the appropriate section before selecting a response.

Materials & experimental systems

n/a	Involved in the study	
☐	☒	Antibodies
☒	☐	Eukaryotic cell lines
☒	☐	Palaeontology and archaeology
☒	☐	Animals and other organisms
☒	☐	Clinical data
☒	☐	Dual use research of concern
☒	☐	Plants

Methods

n/a	Involved in the study	
☒	☐	ChIP-seq
☒	☐	Flow cytometry
☒	☐	MRI-based neuroimaging

Antibodies

Antibodies used	EPCAM (for epithelial-derived cells at a dilution of 100X, Abcam, Cat#ab223582), CD3 (T cells at a dilution of 50x, Abcam, Cat#ab135372), CD19 (for B cells at a dilution of 50x, Abcam, Cat#ab245235), CD33 (myeloid cells at a dilution of 50x, Abcam,

Cat#ab269456), CD56 (NK cells at a dilution of 50x, Abcam, Cat#ab220360), VEGFC (for endothelial cells at a dilution of 50x, Abcam, Cat#ab83905), a-SMA (cancer-associated fibroblasts at a dilution of 200x, Cell Signaling Technology, Cat#19245). CoraLite488 or CoraLite594 Conjugated Antibodies were used as secondary antibodies.

Validation

All the antibodies were applied on Immunofluorescence, and the species of samples was human.

Plants

Seed stocks

Report on the source of all seed stocks or other plant material used. If applicable, state the seed stock centre and catalogue number. If plant specimens were collected from the field, describe the collection location, date and sampling procedures.

Novel plant genotypes

Describe the methods by which all novel plant genotypes were produced. This includes those generated by transgenic approaches, gene editing, chemical/radiation-based mutagenesis and hybridization. For transgenic lines, describe the transformation method, the number of independent lines analyzed and the generation upon which experiments were performed. For gene-edited lines, describe the editor used, the endogenous sequence targeted for editing, the targeting guide RNA sequence (if applicable) and how the editor was applied.

Authentication

Describe any authentication procedures for each seed stock used or novel genotype generated. Describe any experiments used to assess the effect of a mutation and, where applicable, how potential secondary effects (e.g. second site T-DNA insertions, mosiacism, off-target gene editing) were examined.
